# A case of bilateral acute depigmentation of the Iris in one of two identical twins

**DOI:** 10.1186/s12886-019-1282-z

**Published:** 2020-01-06

**Authors:** Spencer Langevin, Alexandra Gershkovich, Brian P. Marr

**Affiliations:** Harkness Eye Institute, Columbia University Irving Medical Center, New York, NY 10032 USA

**Keywords:** Iris, Depigmentation, Pitcher plant extract, iris transillumination

## Abstract

**Background:**

Bilateral Acute Depigmentation of the Iris (BADI) is a condition which was first described in a case series from Turkey by Tugal-Tutkin and Urgancioglu in (Graefes Arch Clin Exp Ophthalmol 244:742-6, 2006). The condition is characterized by bilateral acute depigmentation and discoloration of the iris stroma, pigment dispersion, and deposition of pigment in the angle. In our case we report a patient who developed BADI after receiving pitcher plant extract injections for chronic migraine, while her identical twin sister has normal iris architecture and pigmentation and never received any pitcher plant injections.

**Case presentation:**

Patient is a 41-year-old female with history of pitcher plant extract injections to her face for chronic migraine, who later developed bilateral depigmentation of the iris. She did not have any signs of anterior segment uveitis or iridocyclitis. She has an identical twin sister who maintained normal iris pigmentation during the entire course.

**Conclusions:**

Bilateral Acute depigmentation of the is a recently discovered condition described in the literature in Turkish patients (Tugal-Tutkun and Urgancioglu, Graefes Arch Clin Exp Ophthalmol 244:742-6, 2006; Tugal-Tutkun et al., Ophthalmology 116(8):1552-7, 2009). This condition affects mainly young females and is characterized by acute bilateral stromal depigmentation, without other pathologic ocular findings. These patients usually maintain normal vision and do not develop significant glaucoma from pigment collecting in the anterior chamber angle. This condition can be mistaken for Fuchs’ heterochromic iridocyclitis, pigment dispersion syndrome, pseudoexfoliation syndrome, and viral iridocyclitis. This is the first reported case in North America and is important for differentiation from the above pathologies. Our patient had a history of pitcher plant extract injections to the face but it is unclear if this is associated with our patient’s development of BADI. As awareness of this condition progresses, a possible etiology may be elucidated.

## Background

Bilateral Acute Depigmentation of the Iris (BADI) is a condition which was first described in a case series from Turkey by Tugal-Tutkin and Urgancioglu in 2006 [1]. The condition is characterized by bilateral acute depigmentation and discoloration of the iris stroma, pigment dispersion, and deposition of pigment in the angle. This etiology of this condition is unknown, and only a few cases have been reported in the literature. The initial cases of BADI were reported in Turkey [1, 2], however, recently cases have been reported in both Brazil [3] and Egypt [4]. In our case we report a woman who developed BADI after receiving pitcher plant extract injections for chronic migraine, while her identical twin sister has normal iris architecture and pigmentation, and never received any pitcher plant extract injections.

## Case presentation

**Fig. 1:**
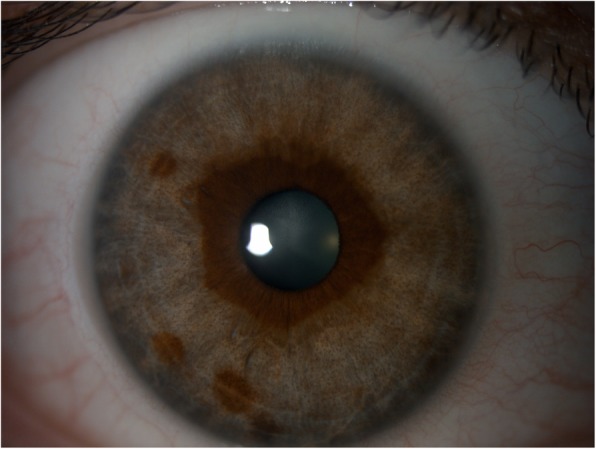
Depigmentation of the iris stroma of the right eye with preservation of stromal pigment within 2 mm of the pupillary margin

**Fig. 2:**
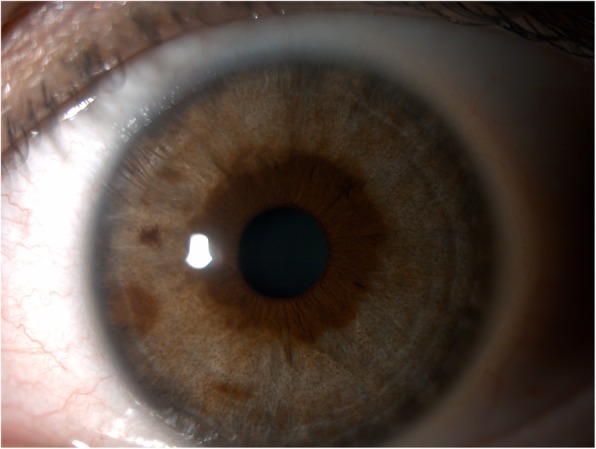
Depigmentation of the iris stroma of the left eye with preservation of stromal pigment within 2 mm of the pupillary margin

**Fig. 3:**
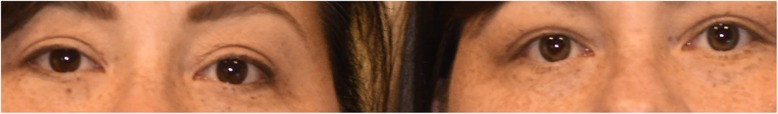
Depigmentation of the iris of both eyes in one twin sister (on the right) compared to normal pigmentation of the other twin sister (on the left)

Our patient is a 41-year-old white female with past medical history significant for psoriasis, temporomandibular joint pain, hiatal hernia, and migraine for which she received pitcher plant extract injections at multiple sites in her face and head every month for approximately 12 months prior to initial presentation. She was in her usual state of health when her identical twin sister noticed that her irides had changed color, prompting her to seek evaluation. She presented to our clinic with a best corrected vision of 20/25 in the right eye and 20/20 in the left eye. Intraocular pressures were 20 mmHg in both eyes. Eyelids, lacrimal system, and adnexa were normal in both eyes. Conjunctiva and cornea were clear in both eyes, and no keratic precipitates were seen. There was no anterior chamber cell or flare in either eye, and there was no posterior synechiae. Bilateral, symmetric, depigmentation of the peripheral iris stroma was present without transillumination defects (see Figs. 1 and 2). She had pigmentary sparing along the pupillary margin in both eyes with no pigment changes in the several overlying iris nevi and freckles. There was noted to be clump-like dusting of pigment throughout iris stroma of both eyes. Gonioscopy revealed open angles in both eyes without evidence of deep pigmentation, peripheral anterior synechiae or segmental pigment deposition. Dilated fundus exam revealed normal pigmentation in both eyes, and was otherwise unremarkable. The patient has been followed for approximately 5 years and her irises have remained depigmented. Her identical twin still has no signs of depigmentation of her iris and maintains brown irides at last exam 1 month ago (see Fig. 3).

## Conclusions

Bilateral Acute depigmentation of the iris is a recently discovered condition described in the literature in Turkish patients [1, 2]. This condition affects mainly young females, and is characterized by acute bilateral stromal depigmentation without other pathologic ocular findings. These patients usually maintain normal vision and do not develop significant glaucoma from pigment collecting in the anterior chamber angle. This condition can be mistaken for Fuchs’ heterochromic iridocyclitis, pigment dispersion syndrome, pseudoexfoliation syndrome, and viral iridocyclitis due to varicella zoster virus, herpes simplex Virus, and cytomegalovirus. Fuchs’ heterochromic iridocyclitis is characterized by unilateral presentation in all but a few cases, and is also characterized by white-stellate keratic precipitates and low-grade anterior chamber inflammation [5], which our patient did not have. Pigment dispersion syndrome, as well as pseudoexfoliation, are conditions characterized by loss of pigment from the posterior iris pigmented epithelium, with transillumination defects, and accumulation of pigment in the anterior chamber angle, along the zonules and on the anterior lens capsule [6], all of which were absent in our patient as well. Herpetic iridocyclitis is almost always unilateral, and accompanied by eye pain, redness, photophobia, anterior chamber inflammation, hyphema, keratic precipitates, posterior synechiae, decreased corneal sensation, iris atrophy, irregular pupil, transillumination defects, and elevated intraocular pressure [7]. Our patient did not display any of these features on exam.

Bilateral Acute Iris Transillumination (BAIT) is another recent entity described in the literature as a condition with acute onset of bilateral iris transillumination defects, with loss of associated iris pigment epithelium after using fluoroquinolones and other antibiotics [8]. In our case, our patient did not have transillumination defects and did not take antibiotics before onset of her depigmentation.

Upon review of the cases of BADI, including ours, we noted one important clinical feature which is a maintenance of iris pigmentation within 1-2 mm of the pupil margin. The majority of cases presented in the literature had this common clinical feature [1–4]. BADI is also easily mistaken for iridocyclitis but given the absence of uveitis symptoms and a different pattern of depigmentation, it is a condition which should be readily made clinically. The etiology for such a condition is not well known as only 38.5% of patients in the review by Tugal-Tutkin had a viral prodrome before developing the condition [2]. In our patient she denied any viral prodrome but she reported monthly injections of pitcher plant extract for chronic migraine for a total of 12 months. The pitcher plant toxin has been extensively studied for its analgesic effects. It acts upon the C fibers of nerves and contains an unidentified toxin that potentiates action of ammonium ions [9], resulting in decreased pain. This injection has never been reported to cause any complications. The effect of pitcher plant extract on iris pigmentation is unknown, but the timing of the treatment and change in iris color suggests a relationship, though coincidence may be the ultimate explanation.

Reports have shown that patients with this condition can have spontaneous re-pigmentation as well, however this has not yet been noted in our patient who we have been following for several years.

Our case is significant as it is the first reported in the Western Hemisphere, as well as the first case amongst identical twins. As awareness of this condition increases, an etiogenesis may be discovered as more cases are studied.

## Data Availability

Not applicable.

## Abbreviations

BADI: Bilateral Acute Depigmentation of the Iris
BATI: Bilateral Acute Iris Transillumination
